# The Hematological and Biochemical Effects from Pesticide Exposure on Thai Vegetable Farmers

**DOI:** 10.3390/toxics11080707

**Published:** 2023-08-17

**Authors:** Siriphan Bunsri, Nutnichawan Muenchamnan, Warangkana Naksen, Parichat Ong-Artborirak

**Affiliations:** 1 Faculty of Public Health, Chiang Mai University, Chiang Mai 50200, Thailand; siriphan_bunsri@hotmail.com (S.B.); nutnichawan@gmail.com (N.M.); 2 Department of Research and Medical Innovation, Faculty of Medicine Vajira Hospital, Navamindradhiraj University, Bangkok 10300, Thailand

**Keywords:** pesticide, herbicide, insecticide, farmers, blood, liver, kidney

## Abstract

Pesticide-related health concerns are a global public health issue. Few studies in Thailand have explored the hematological and biochemical effects of occupational pesticide exposure. The goal of this study was to investigate the effects of pesticides on the hematology, hepatic, and renal function of Thai vegetable farmers. A cross-sectional study was carried out in Chiang Mai, northern Thailand. A total of 124 apparently healthy vegetable farmers were interviewed about their lifetime exposure to agricultural pesticides. Blood samples were collected via venipuncture to be tested for complete blood count (CBC), liver function, and kidney function. Approximately 46% of the farmers were pesticide users who reported a history of pesticide use for their crops, while 54% were non-pesticide users. In the male farmers, the activities of aspartate aminotransferase (AST), mean corpuscular hemoglobin (MCH), and mean corpuscular hemoglobin concentration (MCHC) were significantly higher in the pesticide users compared to the non-pesticide users, while the estimated glomerular filtration rate (eGFR), hematocrit (HCT), and red blood cells (RBC) were significantly lower (*p* < 0.05). In the females, the pesticide users had significantly higher levels of alanine aminotransferase (ALT), alkaline phosphatase (ALP), and MCHC than the non-pesticide users (*p* < 0.05). Pesticide use among Thai vegetable farmers may cause hematological alterations and increase the risk of hepatic and renal dysfunction. Some hematological and biochemical parameters may be used for monitoring to protect them from the adverse health effects of occupational exposure to pesticides.

## 1. Introduction

Pesticides are still widely used in agriculture around the world. Thailand is an agricultural country that imports a large amount of chemicals, mostly glyphosate for herbicides and abamectin for insecticides [1]. Illegal and excessive pesticide use, as well as poor exposure prevention behaviors, such as using personal protective equipment (PPE) during pesticide application, have been noted among Thai farmers, particularly those who grow vegetables [2,3]. The use of pesticides can have both acute and chronic negative effects on human health, with a direct impact on farmers, and can also cause ecosystem damage, mainly affecting animals [4,5,6]. It is estimated that about 44% of farmers are poisoned by pesticides annually [7]. Exposure to pesticides may cause oxidative stress and DNA damage [8,9]. Many studies have also found an increased risk of cancers such as non-Hodgkin lymphoma, leukemia, brain, prostate, kidney, and liver [10,11,12,13,14].

Exposure to different pesticides may impact organ functions due to their cytotoxic effects, and multiple biomarkers may be used to monitor the early adverse effects of pesticides [15,16]. Numerous studies have revealed that individuals who are exposed to pesticides experience significant hemotoxic effects [16,17,18,19,20,21,22]. A study found significant hematological changes in Thai rice farmers [23]. However, little is known about the hematological effects of pesticide exposure in Thailand.

In addition, the effects of pesticides may include liver and kidney dysfunction, as measured by blood biochemical parameters. Many studies have found changes in liver function indicators, such as serum aspartate aminotransferase (AST), alanine aminotransferase (ALT), and alkaline phosphatase (ALP), associated with occupational pesticide exposure [15,16,20,22,24,25]. Alterations in these enzyme activities among Thai pesticide-using farmers are unclear due to previous studies yielding insignificant results [23,26,27].

According to literature reviews, pesticide exposure is a risk factor for the development of nephrotoxic effects, which include diminished renal function and chronic kidney disease (CKD) [28,29,30,31]. Exposure to certain pesticides may also increase the risk of end-stage renal disease [32]. Several studies have observed associations between exposure to herbicides and insecticides, including carbamates and organophosphates, and a lower estimated glomerular filtration rate (eGFR) [33,34,35,36]. Kidney function alterations have been reported in Thai farmers who use organophosphates and carbamates, depending on their use of PPE [37].

As evidence from research in Thailand is limited, this study was conducted to investigate the effects of pesticides on the hematological and biochemical parameters in vegetable farmers. The findings may be useful for the surveillance and prevention of pesticide-related adverse health effects among Thai farmers.

## 2. Materials and Methods

**Figure 1:**
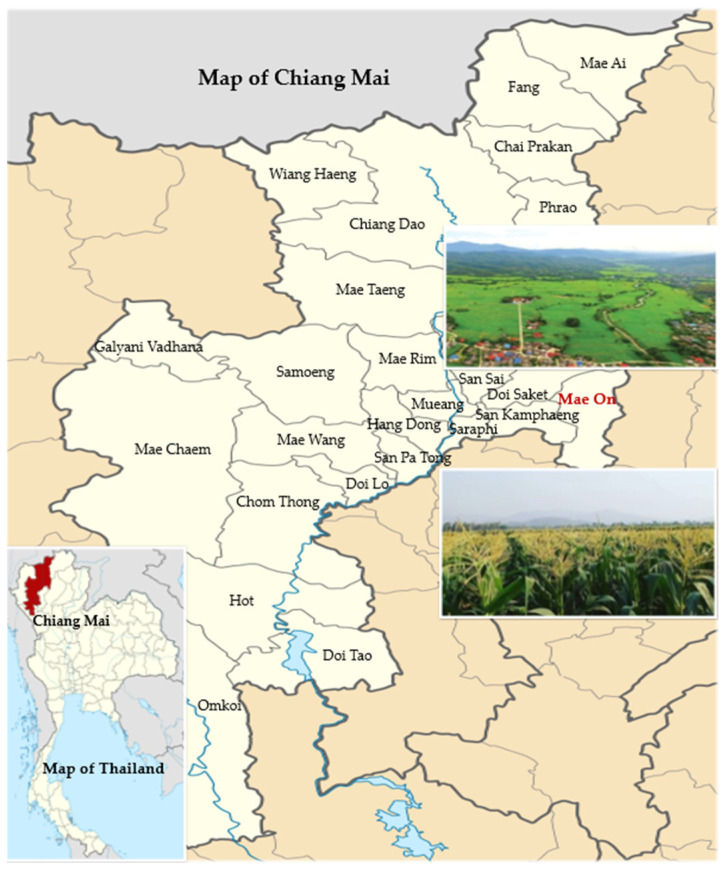
Location of study area in Mae On District in Chiang Mai, Thailand. Adapted from https://commons.m.wikimedia.org/wiki/File:Thailand_Chiang_Mai_locator_map.svg, (accessed on 31 July 2023).

This cross-sectional study was conducted on farmers from the Mae On District in the Chiang Mai Province, northern Thailand (Figure 1). Both conventional and organic farmers are located in this agricultural region. The sample size for the study was calculated using the G*Power program for two mean independent comparisons, with a medium effect size of 0.5, power of 80%, and alpha of 5%. We increased the sample size to 25%, totaling 128 farmers. The inclusion criteria included being a male or female vegetable farmer between the ages of 18 and 65, having worked for at least a year, being free of any underlying medical conditions (such as hypertension, diabetes, cirrhosis, or kidney failure), not being pregnant, and being able to communicate in Thai. Convenience sampling was used to recruit study participants who were eligible through public relations by village health volunteers. Due to a sample loss during the data collection, we obtained completed data for 124 farmers at the end. This study protocol was approved by the Committee of Research Ethics, Faculty of Public Health, Chiang Mai University (Document No. ET004/2021). Prior to the data collection, all the study participants provided written informed consent.

The data were collected in 2021 between February and March. A face-to-face interview with the vegetable farmers was conducted by trained research assistants using a questionnaire. The questionnaire contained demographic variables (e.g., gender, age, body mass index, marital status, education, monthly family income, and distance from home to nearest farm), work-related information (e.g., work experience, farm size, and types of vegetables cultivated), health behaviors (e.g., smoking, drinking alcohol, caffeine consumption, supplement use, exercise, food consumption, and daily water consumption), and a question about their lifetime history of mixing or applying agricultural pesticides (ever-/never-use), which showed a good reliability of self-reported information on pesticide use [38]. The farmers were divided into two groups: pesticide users who reported having used pesticides on their crops and non-pesticide users who had never used pesticides. The pesticide users were also asked for additional information on the duration of their pesticide use, pesticide application, pesticide types and trade names, use of personal protective equipment (PPE), keeping and cleaning of agricultural equipment and pesticide containers, and pesticide container disposal.

Blood sampling was performed by nurses via venipuncture in the morning to evaluate the hematological and biological parameters. The blood samples were then transferred to the laboratory of Bangkok RIA Lab Co., Ltd. (Medical Technology Clinic) Chiang Mai Branch located in Chiang Mai, Thailand, which is certified by the Bureau of Laboratory Quality Standards, Ministry of Public Health. The hematological parameters from the complete blood count (CBC) tests included white blood cells (WBC), hemoglobin (HGB), hematocrit (HCT), mean corpuscular volume (MCV), mean corpuscular hemoglobin (MCH), mean corpuscular hemoglobin concentration (MCHC), red blood cells (RBC), and platelets (PLT). The liver function test measured the serum AST, ALT, and ALP. To assess the kidney function, the eGFR was calculated from the creatinine using the CKD-EPI Equation for Whites [39].

The SPSS statistical program (SPSS Inc., Chicago, IL, USA) was used to analyze the data. The results of the study were described using frequency (n), percentage (%), arithmetic mean, standard deviation (SD), and median. The Chi-square test was used to compare the categorical variables, including the demographic characteristics, work-related information, and health behaviors between two farmer groups: pesticide users and non-pesticide users. The independent *t*-test and Mann–Whitney U test were used to compare differences in the continuous outcomes, such as the hematological and biological parameters for normal and non-normal data distributions, respectively, between the two farmer groups. A *p*-value of 0.05 was considered to be statistically significant. In this study, the *p*-values were not adjusted for multiple comparisons to avoid rejecting the null hypothesis, as this may lead to fewer errors of interpretation when the data are actual observations on nature, whereas an adjustment is required in confirmatory studies [40,41].

## 3. Results

Of the 124 vegetable farmers, 57 (46.0%) were pesticide users who reported a history of using pesticides for their crops, while 67 (54.0%) were non-pesticide users. The personal characteristics and work-related information of the two groups of farmers are shown in Table 1. It was found that the pesticide and non-pesticide users had significantly different sex proportions (*p* < 0.001). There were no differences in terms of proportions of age, body mass index, marital status, education, monthly family income, farm proximity, work experience, farm size, growing babycorn, and other vegetable cultivation between the two groups of farmers. In terms of health, a significant difference in the proportion of smokers was observed between the farmers who used pesticides and those who did not (*p* < 0.001), while there was no difference between the two groups in terms of the following health behaviors: alcohol consumption (no/sometimes, always), caffeine drinking (no/sometimes, always), supplement use (no, yes), exercise (no, yes), eating salty foods (very salty, moderately salty, less salty), plant food consumption (occasionally, more than once a week), meat consumption (rarely, every week, every day), and daily water consumption (<5 glasses, 5–7 glasses, 8–10 glasses, 11–13 glasses, >13 glasses). Because of the differences in sex proportions between the two groups, smoking was also observed to be different. As a result, subanalyses of the blood outcomes based on sex were performed.

The pesticide users were 61.4% male (*n* = 35) and 38.6% female (*n* = 22). The average duration of pesticide use was 12.2 ± 10.1 years (14.1 ± 10.8 years for males and 9.1 ± 8.3 years for females). There were 89.5% mixers (91.4% for males and 86.4% for females) and 89.5% sprayers (94.3% for males and 81.8% for females). The history of the individual pesticides used by the farmers is shown in Table 2. Paraquat (24.6%) and glyphosate (19.3%) were found to be the herbicides most commonly used by the farmers. Acetamiprid (26.3%) and carbaryl (21.0%) were the most commonly reported insecticides. Approximately 47.4% of the farmers always used all types of PPE when applying pesticides, including hats (93.0%), chemical respirators (49.1%), gloves (91.3%), long-sleeved shirts and long pants (100%), and boots (96.5%). Most kept their agricultural equipment and pesticides in a space separate from their home (54.4%), cleaned their agricultural equipment and containers on the farm (68.4%), and disposed of the pesticide packaging in a landfill (64.9%).

More than 90% of the vegetable farmers had CBC, ALP, AST, ALT, and eGFR values within the normal range. Comparisons of the hematological and biochemical parameters between the pesticide users and non-users are shown in Table 3 and Table 4. In the males, the mean HCT and RBC were significantly lower in the pesticide users compared to the non-users (*p* < 0.05). There were differences in the medians of the following outcomes between the pesticide users and non-users: MCH (28.7 vs. 27.55 pg), MCHC (32.6 vs. 31.3 g/dL), eGFR (102.55 vs. 112.58 mL/min/1.73 m^2^), and AST (27 vs. 19 units/L). In the females, the pesticide users had a significantly higher mean ALP than the non-users (*p* < 0.05). The pesticide users and non-users also had different medians for MCHC (31.2 vs. 30.9 g/dL) and ALT (19 vs. 15 units/L).

## 4. Discussion

This study demonstrated the hematological and biochemical effects from pesticide exposure on Thai vegetable farmers. In terms of the hematological parameters, the pesticide users had lower HCT and RBC values, higher MCH and MCHC values in the male farmers, and only lower MCHC values in the female farmers. This may have been due to pesticide exposure altering iron homeostasis, which affects the red blood cells [42]. Long-term exposure could disrupt the balance between free radical production and antioxidant defenses, resulting in DNA damage [20]. This significant difference is consistent with previous findings for pesticide-exposed people, such as male pesticide sprayers and agricultural workers, including greenhouse workers [16,17,18,19,20,21,22]. In Thailand, a study found that rice farmers had significantly lower MCH and MCHC values [23], whereas other studies have found no difference in the CBC parameters for pesticide-using farmers [26,27]. These inconsistent results may be due to differences in the pesticide type, frequency, and duration in each study, as well as differences in individual immune responses [31]. Our findings indicated a higher risk of blood disorders such as anemia for vegetable farmers who are repeatedly exposed to pesticides, particularly men.

In our study, the pesticide users had significantly higher levels of serum enzymes that indicate hepatic damage, including AST for males and ALT and ALP for females. Our results are consistent with the findings of earlier studies, which have found elevated levels of these enzyme activities in farmers and agricultural workers, including pesticide sprayers [15,16,20,24,25,43]. High ALT and AST levels may indicate lysis of the liver cells and leakage of enzymes into the blood, causing cytotoxic effects [25]. Elevated ALP levels may be caused by an increased plasma membrane permeability or cellular necrosis [16,44]. Pesticide exposure, such as to organophosphate, may disrupt the liver metabolism and mitochondrial metabolic pathways caused by oxidative damage [45]. According to Khan et al., (2008) [15], pesticide residues such as methomyl, imidacloprid, cypermethrin, and methamidophos were positively correlated with ALT or AST in pesticide-exposed tobacco farmers. In line with these findings, the vegetable growers in our study were also exposed to these pesticides. Even though previous studies on Thai orchid farmers and rice farmers who used pesticides observed no difference in liver function tests such as AST, ALT, and ALP [23,26,27], our findings indicated the potential effects of pesticide exposure on hepatotoxicity among vegetable farmers.

In terms of renal function, eGFR was found to be lower in the male farmers who used pesticides. This significant difference was in agreement with previous findings for pesticide-exposed individuals, in which associations with lower eGFR were observed for organophosphates and carbamates, as well as herbicides such as atrazine and glyphosate [33,34,35,36]. Organophosphate exposure may alter the glomeruli, as well as the proximal and distal tubules [46,47]. Neonicotinoids have been identified as a possible risk factor for renal tubular dysfunction [48]. Renal tubule injury, resulting in inflammation and fibrosis, may also lead to the progression of CKD [49]. Previous research has found that lifetime exposure to pesticides such as glyphosate, paraquat, and organophosphates in paddy fields was associated with CKD in farmers [28]. Among Thai farmers, exposure to organophosphates and carbamates, as well as PPE use, were significantly associated with an increased risk of CKD [37]. This indicates that the farmers investigated in our study may be at a higher risk of renal injury as a result of not always using PPE, in particular chemical respirators. Another study found that glyphosate use and the intensity of exposure were related to serum creatinine, but not to eGFR [50]. Some pesticides used by farmers in this study, such as alachlor, atrazine, glyphosate, paraquat, and permethrin, have been linked to CKD [51]. According to our findings, long-term pesticide exposure may cause nephrotoxic changes and increase the risk of CKD in vegetable farmers.

In addition, these findings emphasize the importance of strict pesticide legislations and preventive measures for Thai farmers. In comparison to other countries, the pesticide regulation in Thailand is characterized by the lack of a consolidated, uniform system designed specifically for pesticide management, which has weakened the enforcement of the current regulations, resulting in pesticide misuse/overuse [2]. Regarding the study’s limitations, the cross-sectional design may not be able to draw a causal relationship between pesticide exposure and changes in the hematological and biochemical parameters. A larger sample size is required to increase the power of the analysis, as the data were subanalyzed based on gender. The study did not look into specific pesticides and the intensity of exposure. A pesticide residue analysis to assess internal exposure, such as urinary metabolites, is recommended for future research. Other factors, such as work clothing and heat stress, may also be considered. However, our findings add to the evidence on the effects of pesticide exposure on hematological and biochemical alterations among Thai farmers.

## 5. Conclusions

Our findings emphasized that occupational exposure to pesticides may alter hematological and biochemical parameters, reflecting hemotoxic, nephrotoxic, and hepatotoxic effects among Thai vegetable farmers who mixed or sprayed pesticides. These parameters might be useful in tracking the early negative effects of pesticides on Thai farmers’ health. An intensive intervention to provide knowledge about the potential risks of occupational pesticide exposure and promote the appropriate use of PPE, particularly chemical respirators, should be considered to minimize pesticide exposure and prevent health effects in Thai farmers. Moreover, pesticide-related problems must be recognized and prioritized by the government in order to develop effective policies and strategies, as well as to continue extremely strict actions for strengthening the weak legislation and regulations, ultimately leading to an actual reduction in these problems.

## Figures and Tables

**Table 1:** Demographic and work characteristics of pesticide and non-pesticide users among vegetable farmers (*n* = 124).

Factor	Pesticide Users (*n* = 57)	Non-Users (*n* = 67)	*p*-Value
Sex			<0.001
Male	35 (61.4%)	12 (17.9%)	
Female	22 (38.6%)	55 (82.1%)	
Age			0.153
≤35 years	5 (8.8%)	13 (19.4%)	
36–55 years	34 (59.6%)	40 (59.7%)	
>55 years	18 (31.6%)	14 (20.9%)	
Body mass index (BMI)			0.261
Underweight (<18.5 kg/m^2^)	1 (1.8%)	2 (3.0%)	
Normal (18.5–22.9 kg/m^2^)	21 (36.8%)	25 (37.3%)	
Overweight (23.0–29.9 kg/m^2^)	21 (36.8%)	15 (22.4%)	
Obese (≥30 kg/m^2^)	14 (24.6%)	25 (37.3%)	
Marital status			0.079
Single/Divorced/Widowed	6 (10.5%)	15 (22.4%)	
Married	51 (89.5%)	52 (77.6%)	
Education			0.129
Primary school	35 (61.4%)	32 (47.8%)	
Secondary school or higher	22 (38.6%)	35 (52.2%)	
Monthly family income			0.816
<10,000 Baht (<285 USD)	25 (43.9%)	28 (41.8%)	
≥10,000 Baht (≥285 USD)	32 (56.1%)	39 (58.2%)	
Farm proximity			0.489
<300 m.	23 (40.4%)	23 (34.3%)	
>300 m.	34 (59.6%)	44 (65.7%)	
Work experience			0.079
≤5 years	11 (19.3%)	23 (34.3%)	
6–10 years	9 (15.8%)	14 (20.9%)	
11–20 years	13 (22.8%)	15 (22.4%)	
≥21 years	24 (42.1%)	15 (22.4%)	
Farm size			0.170
<8000 m^2^	27 (47.4%)	40 (59.7%)	
≥8000 m^2^	30 (52.6%)	27 (40.3%)	
Growing babycorn			0.186
No	23 (40.4%)	35 (52.2%)	
Yes	34 (59.6%)	32 (47.8%)	
Other vegetable cultivation			0.389
No	22 (38.6%)	31 (46.3%)	
Yes	35 (61.4%)	36 (53.7%)	
Growing rice			0.252
No	40 (70.2%)	53 (79.1%)	
Yes	17 (29.8%)	14 (20.9%)	

**Table 2:** History of individual pesticide use among vegetable farmers.

Chemical Class	Common Name	WHO Class	Pesticide Users (*n* = 57)
Herbicide			30 (52.6%)
Bipyridylium	Paraquat	II	14 (24.6%)
Glycine	Glyphosate	III	11 (19.3%)
Phenoxy	2,4-D	II	3 (5.3%)
Chloroacetamide/Anilide	Alachlor	II	1 (1.8%)
Triazines	Atrazine	III	1 (1.8%)
Insecticide			48 (84.2%)
Organophosphate	Chlorpyrifos	II	1 (1.8%)
	Methamidophos	Ib	1 (1.8%)
	Dichlorvos	Ib	1 (1.8%)
	Profenofos	II	1 (1.8%)
Carbamate	Carbaryl	II	12 (21.0%)
	Methomyl	Ib	8 (14.0%)
	Carbofuran	Ib	3 (5.3%)
	Carbosulfan	II	2 (3.5%)
Neonicotinoid	Acetamiprid	II	15 (26.3%)
	Imidacloprid	II	8 (14.0%)
	Dinotefuran	III	1 (1.8%)
Avermectin	Abamectin	Ib	7 (12.3%)
	Emamectin	II	4 (7.0%)
Pyrethroid	Cypermethrin	II	4 (7.0%)
Phenylpyrazole	Fipronil	II	5 (8.8%)
Spinosyn	Spinetoram	U	4 (7.0%)

Note: Ib = Highly hazardous; II = Moderately hazardous; III = Slightly hazardous; and U = Unlikely to present acute hazard in normal use.

**Table 3:** Hematological parameters between the two groups of vegetable farmers.

Outcome		Pesticide Users (*n* = 57)	Non-Users (*n* = 67)	*p*-Value
		Mean (SD)	Mean (SD)	
WBC (mm^3^)	All	6979 (1596)	6671 (1669)	0.299 ^a^
	Male	7021 (1612)	6818 (1323)	0.696 ^a^
	Female	6912 (1605)	6639 (1744)	0.528 ^a^
HGB (g/dL)	All	13.76 (1.56)	12.92 (1.71)	0.005 ^a^
	Male	14.44 (1.20)	14.73 (1.42)	0.497 ^a^
	Female	12.69 (1.48)	12.52 (1.51)	0.666 ^a^
HCT (%)	All	42.84 (4.37)	41.64 (5.10)	0.166 ^a^
	Male	44.51 (3.45)	47.08 (4.17)	0.040 ^a^
	Female	40.18 (4.44)	40.46 (4.50)	0.810 ^a^
MCV (fL)	All	84.91 (10.91)	81.22 (12.44)	0.149 ^b^
	Male	87.76 (9.33)	83.08 (13.07)	0.294 ^b^
	Female	80.36 (11.88)	80.81 (12.39)	0.588 ^b^
MCH (pg)	All	27.31 (4.05)	25.24 (4.07)	0.005 ^b^
	Male	28.56 (3.60)	26.01 (4.20)	0.047 ^b^
	Female	25.31 (4.00)	25.08 (4.06)	0.822 ^b^
MCHC (g/dL)	All	32.11 (1.29)	31.05 (0.67)	<0.001 ^b^
	Male	32.48 (1.34)	31.29 (0.48)	0.017 ^b^
	Female	31.53 (0.96)	31.00 (0.69)	0.014 ^b^
RBC (10^6^/mm^3^)	All	5.10 (0.62)	5.18 (0.70)	0.502 ^a^
	Male	5.12 (0.66)	5.74 (0.60)	0.006 ^a^
	Female	5.08 (0.57)	5.06 (0.67)	0.942 ^a^
PLT (mm^3^)	All	249,947 (58,755)	267,567 (58,711)	0.098 ^a^
	Male	234,971 (49,762)	240,417 (30,485)	0.724 ^a^
	Female	273,773 (65,024)	273,491 (61,839)	0.986 ^a^

Abbreviations: WBC, white blood cell; HGB, hemoglobin; HCT, hematocrit; MCV, mean corpuscular volume; MCH, mean corpuscular hemoglobin; MCHC, mean corpuscular hemoglobin concentration; RBC, red blood cell; and PLT, platelets. ^a^ Independent *t*-test; ^b^ Mann–Whitney U test.

**Table 4:** Liver and kidney function tests between the two groups of vegetable farmers.

Outcome		Pesticide Users (*n* = 57)	Non-Users (*n* = 67)	*p*-Value
		Mean (SD)	Mean (SD)	
ALP (U/L)	All	73.84 (24.64)	65.30 (20.11)	0.036 ^a^
	Male	71.63 (23.72)	71.83 (14.48)	0.978 ^a^
	Female	77.36 (26.22)	63.87 (20.98)	0.020 ^a^
AST (U/L)	All	29.65 (24.86)	18.85 (8.58)	<0.001 ^b^
	Male	35.91 (29.87)	24.08 (13.88)	0.033 ^b^
	Female	19.68 (5.87)	17.71 (6.58)	0.115 ^b^
ALT (U/L)	All	29.28 (24.06)	19.57 (14.30)	<0.001 ^b^
	Male	35.83 (28.31)	30.17 (20.35)	0.366 ^b^
	Female	18.86 (7.85)	17.25 (11.63)	0.029 ^b^
eGFR (mL/min/1.73 m^2^)	All	102.75 (13.59)	106.54 (11.56)	0.169 ^b^
	Male	99.66 (14.60)	110.00 (11.07)	0.020 ^b^
	Female	107.66 (10.31)	105.79 (11.63)	0.569 ^b^

Abbreviations: ALP, alkaline phosphatase; AST, aspartate aminotransferase; ALT, alanine aminotransferase; and eGFR, estimated glomerular filtration rate. ^a^ Independent *t*-test; ^b^ Mann–Whitney U test.

## Data Availability

The data are available upon request from the corresponding author.
